# Systemic Lupus Erythematosus Vasculitis Causing Perforation Peritonitis in Miliary Tuberculosis: A Disease in Disguise

**DOI:** 10.7759/cureus.81479

**Published:** 2025-03-30

**Authors:** Soumyajit Jana, Monika Gureh, Ankur Cheleng, Ayush Vardhan

**Affiliations:** 1 General Surgery, All India Institute of Medical Sciences, Bhubaneswar, Bhubaneswar, IND

**Keywords:** emergency stoma, intestinal perforation, military tb, small bowel necrosis, systemic lupus erythematosus

## Abstract

Systemic lupus erythematosus is a complex disease to manage and is complicated further by coexisting comorbidities. We present the case of a 25-year-old female patient who arrived at the emergency department with complaints of abdominal pain for three days, accompanied by a history of fever and non-bilious vomiting for two days. She had no history of trauma or chronic use of painkillers. She was diagnosed with pulmonary and abdominal tuberculosis (TB) four months previously and has been on anti-tubercular therapy since then. Radiological studies showed a collection with echogenic foci in the right iliac fossa with features of abdominal tuberculosis and pneumoperitoneum likely due to perforation. An emergency laparotomy with end ileostomy and distal mucus fistula was performed, followed by limited ileocecal resection, and the specimen was sent for histopathological examination. Surprisingly, the histopathology study revealed SLE vasculitis, contrary to the initial suspicion of ileocecal TB as the cause of perforation peritonitis. The patient was discharged and was followed up within a week with normal stomal function.

## Introduction

Systemic lupus erythematosus (SLE) is an inflammatory autoimmune multisystem disease. The skin, along with musculoskeletal, hematopoietic, and cardiopulmonary systems, is commonly affected [1]. If untreated, SLE with gastrointestinal involvement can cause perforation and peritonitis, leading to major morbidity as well as mortality in patients. This is due to vasculitis and thrombosis affecting mesenteric vessels of the bowel [2-3].

In SLE, gastrointestinal symptoms are common; though perforation leading to peritonitis is rare, it can have severe, life-threatening complications. This report presents a case of SLE in the background of miliary tuberculosis (TB) with intestinal perforation, where surgical intervention was required and perforation was detected in the base of the cecum near the appendix. Following surgery, the patient suffered from difficult ventilator weaning and surgical site infection (SSI). The patient was treated, discharged, and followed up within a week, showing a functional stoma and granulated wound from a previous midline SSI, which was repaired by secondary suturing. This case highlights how intestinal perforation in SLE patients is life-threatening and requires prompt management.

## Case presentation

A female patient in her 20s presented to the emergency department with complaints of abdominal pain of three days' duration. The patient was apparently fine four days prior; however, one day later, she developed lower abdominal pain and fever that lasted for three days. She also had two episodes of non-bilious vomiting for two days. She had passed stools and flatus the day before she reported to the emergency department. She had no history of trauma, urinary symptoms, or chronic drug usage (painkillers). She was diagnosed with pulmonary and abdominal TB (miliary) four months ago and has been on anti-tubercular therapy (ATT) since then. She has also had systemic lupus erythematosus (SLE) for four years and is currently on hydroxychloroquine. There was no family history of malignancy.

On clinical examination, the abdomen was distended with tenderness and rebound tenderness over the right iliac fossa. Guarding was present with shifting dullness (suggestive of intraperitoneal collection). Bowel sounds were absent. There was a healed lower segment cesarean section scar (Pfannenstiel) present in the lower abdomen. The respiratory system had reduced breath sounds in the left lower regions. No added sounds.

**Figure 1:**
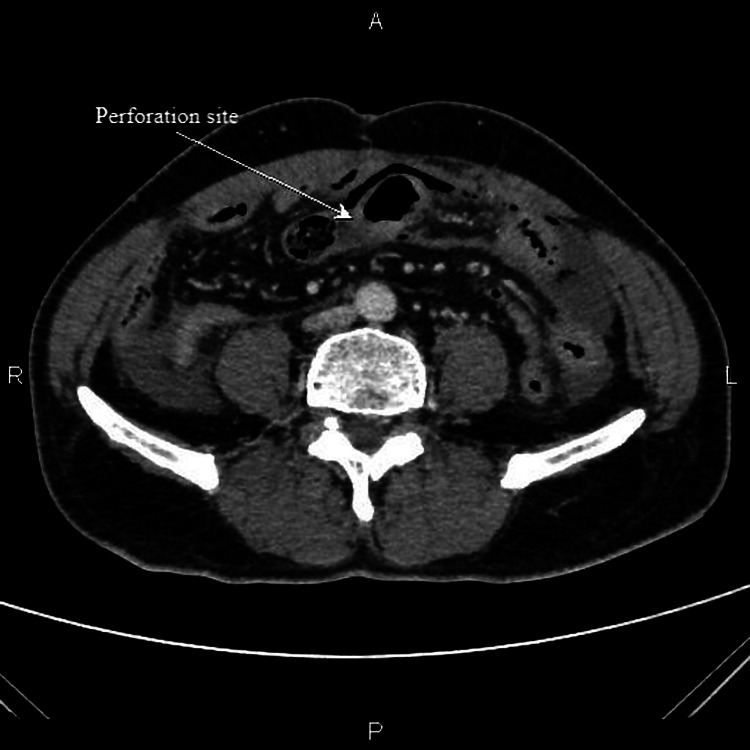
A contrast-enhanced computed tomographic scan (with both intravenous and oral contrast injected) of the abdomen in axial plane showing a defect in bowel wall near the ileocecal junction along with surrounding fat stranding and air foci (marked by arrow). P: posterior, A: anterior, R: right side

On presentation, an immediate abdominal X-ray (erect and supine) was performed, which did not show any gas under the diaphragm. Dilated small bowel loops without any significant air fluid levels were suggestive of small bowel obstruction. Additionally, the chest radiograph was suggestive of pulmonary TB. Ultrasonography (USG) abdomen showed a collection in the pelvis region measuring 6 x 6 cm with echogenic foci and a large ill-defined collection in the right iliac fossa with mild ascites. A contrast-enhanced computed tomography (CECT) scan of the abdomen showed dilatation of jejunal and ileal loops with diffuse bowel wall thickening and edema noted. A defect was noted in the bowel wall near the ileocolic (IC) junction along with surrounding fat stranding, air foci. Pneumoperitoneum was also noted, with partially collapsed proximal transverse colon showing focal wall thickening (Figure 1).

Routine blood parameters were done, where total leukocyte count (tlc): 20K; hemoglobin (hb): 9.8 g/dl and albumin (alb): 1.8 g/dl were the only significant deranged values noted. The other parameters were within normal limits (Table 1). On digital rectal examination, there was no evidence of impacted stools or mass. The rectum was found to be collapsed. On examination, the gloved finger was stained with stool.

**Table 1:** Lab test results on admission *as per the reference range values used in authors' institution (All India Institute of Medical Sciences, Bhubaneswar)

Investigations	Values	Unit	Reference*
Hb	9.8	g/dl	13-17
Tlc	20	*10^3^/cumm	4-11
Alb	1.8	g/dl	3.4-5.4
PH	7.1	-	7.35-7.45
Lactate	3.6	mmol/L	<2

The patient was taken to the emergency operating theatre (OT), where multiple tubercular deposits on the peritoneum and bowel mesentery were found intraoperatively. There was a perforation present at the base of the cecum (Fig. 3). The patient underwent a laparotomy with end ileostomy and distal mucous fistula following a limited ileocecal resection. The resected specimen was sent for histopathological examination.

**Figure 2:**
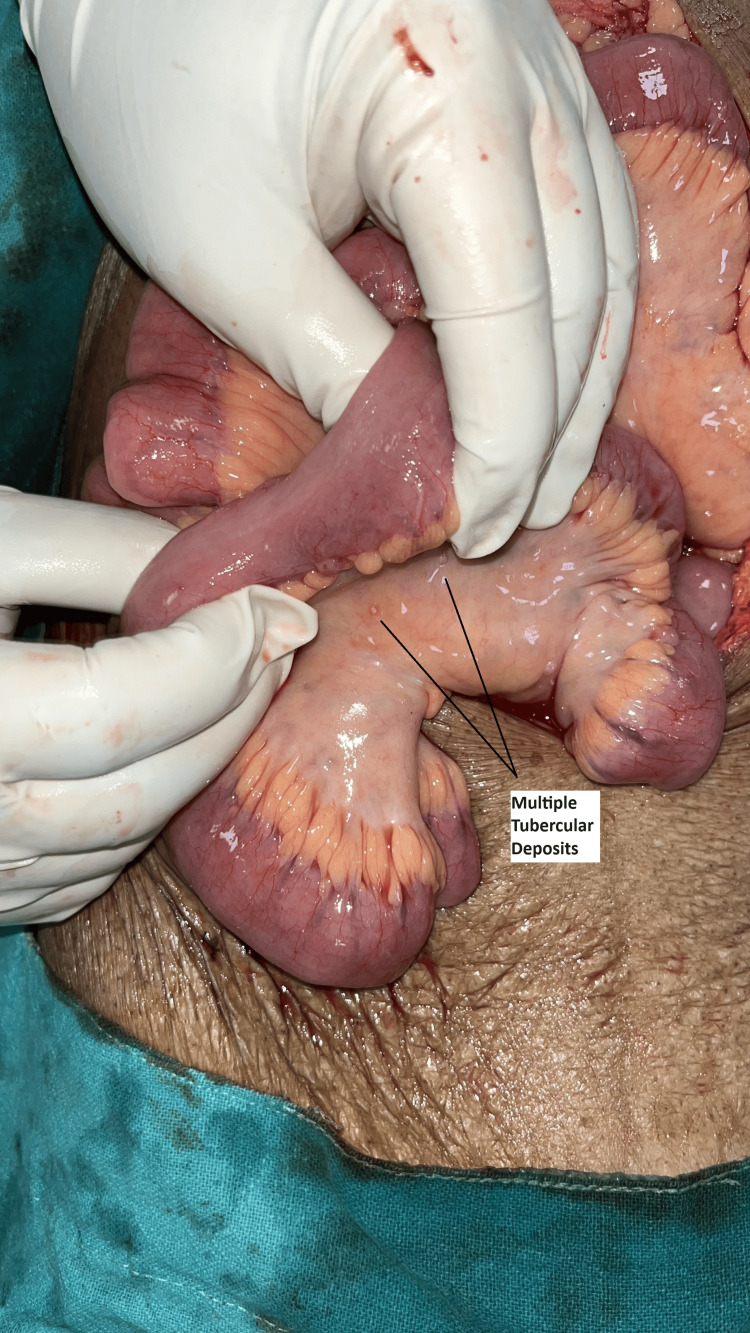
Multiple tubercular deposits in a already known case of TB

**Figure 3:**
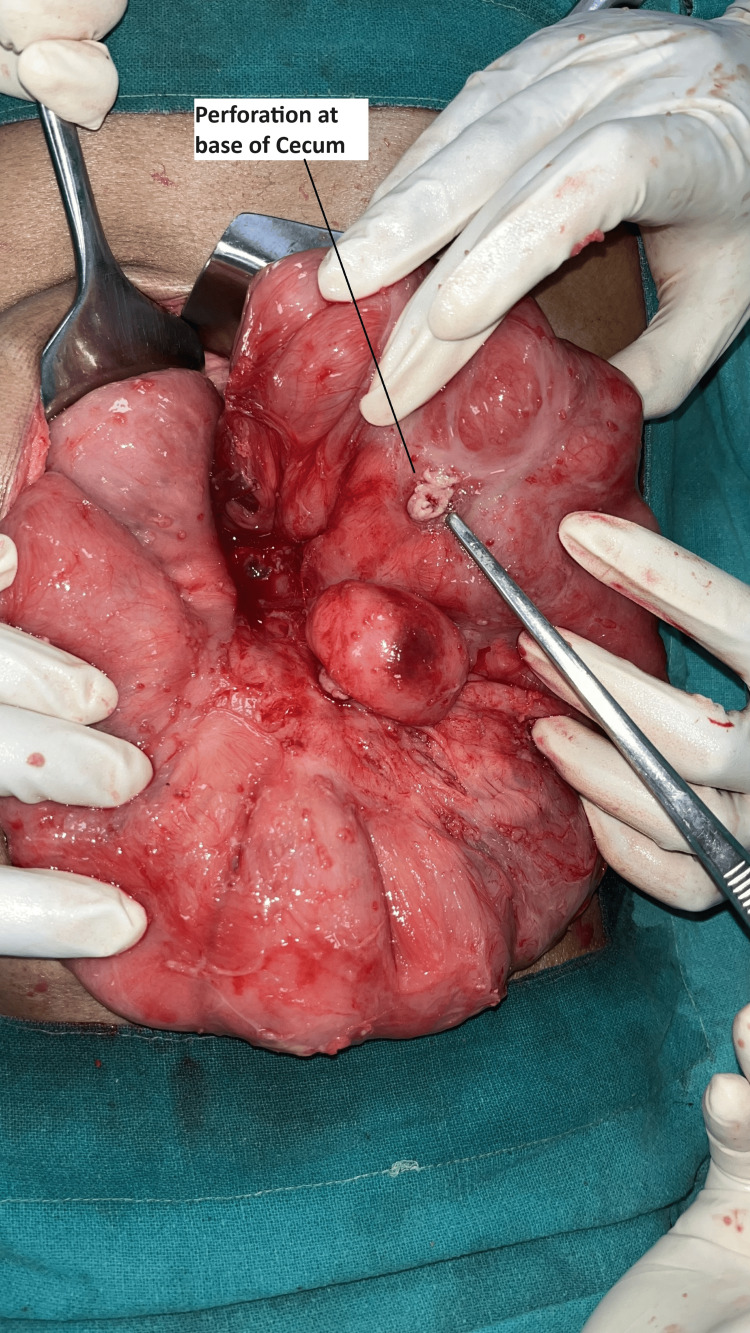
Perforation at the base of cecum

**Figure 4:**
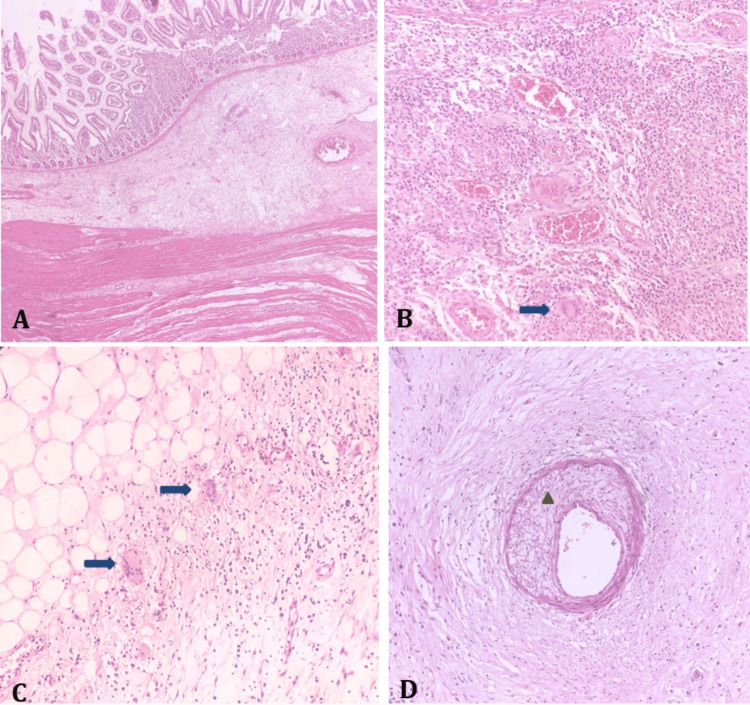
(A) Ileal stricture showing prominent submucosal fibrosis extending into the muscularis propria. (B) Submucosal dense chronic inflammation with non-caseating granulomas and multinulceate giant cells (arrow). (C) Subserosal hyalinisation and inflammation with multinucleate giant cells (arrow). (D) Vessel showing myxoid degeneration of the media (triangle) with perivascular fibroblastic proliferation.

A histopathological examination of the specimen revealed dense submucosal chronic inflammation with non-caseating granulomas and multinucleated giant cells. Subserosal hyalinization and inflammation with multinucleated giant cells were also seen. The vessels exhibited myxoid degeneration of the media with perivascular fibroblastic proliferation. These findings, confirmed by the pathologist, established the diagnosis of SLE-associated vasculitis (Figure 4).

Following surgery, the patient suffered from difficult ventilator weaning, surgical site infection (midline SSI without sheath dehiscence). The patient was treated and discharged from the hospital and was followed up within a week with a functional stoma and granulated wound from a previous midline SSI, which was repaired by secondary suturing.

## Discussion

The clinical symptoms are usually not specific; however, common symptoms are abdominal pain associated with vomiting, diarrhoea, and fever. Lupus enteritis also responds to steroids well. Untreated cases can lead to perforation-induced peritonitis, which increases the overall mortality and morbidity [4-5]. The prognosis is often poor. Hence, early diagnosis and prompt management are a must [6]. Long-term users of corticosteroids and immunosuppressants (which weaken the immune system) are seen in almost all cases of SLE [7]. Any surgical intervention has higher morbidity rates in patients of SLE in comparison to non-SLE patients [8-9].

Initially, the primary goal of treatment includes resuscitation, as patients can present in shock or poor general condition. Further management can be categorised as medical and surgical as follows.

Medical management

A study showed that most patients with SLE vasculitis are treated with hydroxychloroquine (HCQ). Many autoimmune diseases are managed using traditional antimalarial drugs like chloroquine (CQ) and HCQ, which to date are still used to treat patients with SLE. The basic mechanism of these drugs' actions involves inhibiting lysosomal activity, autophagy, pro-inflammatory cytokine signaling and secretion, T-cell proliferation, and toll-like receptor activation [10]. HCQ is usually recommended unless contraindicated. However, high doses can lead to retinopathy and cardiomyopathy. Thus, an HCQ dosage exceeding 5 mg/kg body mass (equivalent to a cumulative dose of >1000 g) is not recommended [10].

A prednisolone dosage of 1 mg/kg/day for treating SLE exacerbations has not been found to be superior to the response obtained from pulse therapy with lower doses of methylprednisolone (0.3 and 0.5 mg/kg/day) [10].

Low glucocorticoid dosage, i.e., less than 5 mg/day of prednisone equivalent, should be considered for long-term glucocorticoid therapy. This is due to the risk of cataracts, ischemic heart disease, osteoporosis, and pathological fractures being common with dosages more than equal to 5mg/day [10].

Surgical management

SLE-induced vasculitis of the gastrointestinal tract is uncommon. The estimated prevalence varies between 0.2 and 14.2% among all diagnosed patients of SLE. Lupus mesenteric vasculitis is the most severe manifestation, with a mortality rate as high as 50%. It may lead to bowel ischemia and potential necrosis of the small or large bowel, which may evolve to perforation and hemorrhage. In most cases, about 80-85%, the superior mesenteric artery is involved. The small bowel is most commonly affected, which includes the ileum and jejunum, whereas the large bowel and rectum are rarely affected. The main symptoms include acute abdominal pain, nausea, vomiting, diarrhea, melena, hematemesis, and bloating. Ascites, though rare, may be present in patients with SLE. Hence, surgical management in patients with severe abdominal pain presenting to the emergency department is almost always needed [11-12].

The SLE disease activity index (SLEDAI) gives an overall prognosis of the disease. In adults with SLE who had intrabdominal vasculitis or thrombosis, there were higher SLEDAI scores, which were associated with surgical abdomens requiring immediate laparotomies. However, this does not exclude the fact that patients with low SLEDAI scores might not require surgeries, although such rare cases are rare. Studies have shown that patients with SLEDAI score > 5 with active disease have a higher prevalence of lupus mesenteric vasculitis than those with inactive disease. Sometimes patients presenting with colitis induced by vasculitis may also require immediate or late laparotomy [12-14].

For SLE vasculitis leading to perforation, the preferred surgical management is stoma formation for initial healing, followed by early or late stoma closure. Alternatively, resection and anastomosis may be considered, with caution due to SLE-related poor wound healing and immunosuppression

Prevalence of TB in SLE

Studies have shown a high disease burden of abdominal TB in SLE. It is most commonly seen in TB-endemic countries. However, the data from these regions remains scarce, underlining the need for the evaluation and implementation of TB prevention strategies and research. Parenteral nutrition still remains one of its key components in treating such patients [15-16].

In a study conducted in Durban, South Africa showed the prevalence of TB in SLE being different in different ethnic groups (12.8%) for Indian people, (15.8%) Black African people and (26.9%) admixed African people; besides establishing the importance of disease activity index as well as use of immunosuppressive therapy being the major risk factors. Another study conducted in China showed the prevalence being only 2.4% (very low), confirming that ethnic factors play an important role in the disease prevalence [17-18]. 

## Conclusions

Perforation peritonitis in patients with SLE presents a significant clinical challenge. Early management can help reduce morbidity and mortality. As prolonged prednisolone use is associated with an increased risk of intestinal obstruction, pulse-dose therapy of prednisolone may help mitigate symptoms. Therefore, careful consideration of prednisolone dosage is essential when managing such patients to minimize the risk of postoperative intestinal obstruction. Early surgical intervention or a timely decision to operate should also be kept in mind while dealing with patients of SLE vasculitis-associated comorbidities like TB, as they contribute to poor prognosis in such patients.
